# Revisiting translator competence in the age of artificial intelligence: the case of legal and institutional translation

**DOI:** 10.1080/1750399X.2024.2344942

**Published:** 2024-04-28

**Authors:** Fernando Prieto Ramos

**Affiliations:** Centre for Legal and Institutional Translation Studies (Transius), Faculty of Translation and Interpreting, University of Geneva, Geneva, Switzerland

**Keywords:** Institutional translation, legal translation, translator competence descriptor, translator training, machine translation, post-editing

## Abstract

Multi-componential models of translation competence are widely used in translator training as a yardstick for curricular and syllabus design. These models must be adapted to reflect professional trends, such as the impact of artificial intelligence, and machine translation in particular, on working methods. This paper describes the process of adapting a pioneering model of legal translation competence to the broader scope of institutional translation in light of recent trends, as verified by triangulating information from multiple interviews, analyses of translation volumes and job descriptors and other professional inputs. The resulting revised descriptor was validated through a survey of 474 translation professionals from 24 international organisations of diverse sizes and domain specialisations. The suitability of the descriptor was corroborated across the board, but variations were found in perceptions of the relevance of sub-competences to ensure translation quality. Profiles with a stronger specialisation in legal translation or more experience in institutional translation showed higher awareness of the relevance of all the sub-competences, especially the core language, strategic and thematic competences, and even more so for translating texts of a legal or administrative nature. The implications of these findings for training purposes in particular are discussed.

## 1. Introduction

The so-called multi-componential models of translation competence currently prevail in translator training as a useful yardstick for curricular and syllabus design, and for the implementation of didactic activities and assessment methods in today’s increasingly learner-centred approaches to training (e.g. González-Davies 2004; Hurtado Albir 2007; Kiraly 2000; Way 2008). Given the practical nature of translation, the breakdown of competences and sub-competences^1^ required of translators establishes a natural bridge between academic and professional requirements in the field. Academic approaches to competence must keep step with the latest trends in professional practice (Gabr 2007; González-Davies and Enríquez-Raído 2016; Gouadec 2007), while practitioners and professional associations can only benefit from the growing wealth of research in the field for purposes such as accreditation, (self-)assessment and continuous professional development.

The question arises of how translation competence models must be adapted to reflect the impact of technological advances, in particular in artificial intelligence and machine translation (MT), on professional practices and translation competence requirements. The work presented here addresses this question in connection with legal and institutional translation as part of the LETRINT project on institutional translation.^2^ The first multi-componential model of its kind in the field of legal translation (Prieto Ramos 2011) has been revised in light of recent trends in international institutional settings, as verified through multiple interviews, analyses of translation volumes and job descriptors and other professional inputs. It has been expanded to take account of all areas of specialisation covered in such settings, and has been validated through a large-scale survey of institutional translation professionals.

The process of updating the translation competence descriptor is presented in Section 3, following a review of existing approaches of specific relevance to legal and institutional translation (Section 2). The survey results are then summarised with a focus on the relevance of the various competence components for institutional translators (Section 4). The implications of these findings for training and for our understanding of the evolving skill-set required of professional translators, within and beyond institutional settings, are discussed in the concluding section.

## 2. Approaches to competence in legal and institutional translation

With the development of Legal Translation Studies (LTS), and particularly in the ‘catalytic’ period between the mid-1990s and the mid-2000s (Prieto Ramos 2014, 270–272), authors in the field increased attention to what is required to achieve proficiency in legal translation. Šarčević (1997), for example, referred to: ‘deep knowledge of legal terminology’, ‘a full understanding of legal reasoning and the ability to solve legal problems, to analyse legal texts, and to foresee how a text will be interpreted and applied by courts’, as well as ‘extensive knowledge of the target legal system and preferably the source legal system’, ‘drafting skills’ and ‘a basic knowledge of comparative law and comparative methods’ (1997, 113–114). Deborah Cao, presupposing that ‘[a] competent legal translator is first of all a competent translator’ (Cao 2007, 40), applied her ‘model of translation proficiency’ (Cao 1996) to legal translation. The author described ‘three sets of variables interacting with one another in the context of situation’ (Cao 2007, 40): (1) translational language competence, including knowledge of legal language; (2) translational knowledge structures, including knowledge of law and legal culture; and (3) translational strategic competence, which ‘is seen as the linkage that relates translational language competence to translational knowledge structures and the features of the context in which translation, and hence interlingual and intercultural communication, takes place’ (Cao 2007, 48).

These contributions can be considered as prelude to the development of structured models specifically devoted to describing the components and specificities of **legal translation competence** (LTC). The first of such models (Prieto Ramos 2011) takes a holistic perspective informed by professional practice and contemporary paradigms of translation competence, particularly those put forward by the PACTE Group (PACTE 2000, 2003, 2005), Kelly (2002) and the European Master’s in Translation (EMT Expert Group 2009). The components of LTC are summarised as follows (Prieto Ramos 2011, 12):

- *Strategic or methodological competence*, which controls the application of the other skills and comprises: analysis of translation briefs, macro-contextualisation and general work planning, identification of problems and implementation of transfer strategies (translation procedures), decision-making argumentation, self-assessment and quality control.
- *Communicative and textual competence*: linguistic, sociolinguistic and pragmatic knowledge, including knowledge of linguistic variants, registers, specialised legal linguistic uses and legal genre conventions.
- *Thematic and cultural competence*: knowledge of legal systems, hierarchy of legal sources, branches of law and main legal concepts; awareness of asymmetry between legal notions and structures in different legal traditions.
- *Instrumental competence (documentation and technology)*: knowledge of specialised sources, information and terminology management, use of parallel documents, application of computer tools to translation.
- *Interpersonal and professional management competence*: teamwork, interaction with clients and other professionals, knowledge of the legal framework for professional practice and fiscal obligations, deontological aspects.

Thematic competence, which is essential to comparative legal analysis for translation decision-making, is considered a distinctive feature of LTC, together with other elements of legal science and legal linguistic knowledge that are integrated in the other sub-competences (e.g. knowledge of legal genres or specialised legal sources as part of textual and instrumental competences).

In turn, subsequent LTC models have, to varying degrees, drawn on the above descriptors to propose similar approaches or applications targeted to specific scenarios. For instance, Piecychna (2013) focuses on hermeneutical aspects and distinguishes between psychological, thematic, textual, and linguistic sub-competences. The first of these sub-competences integrates core strategic and service provision components, such as ‘the ability to identify and solve problems with appropriate strategies and techniques’ and ‘the ability to analyze and *interpret* texts’ (Piecychna 2013, 153). Soriano Barabino (2016, 148–150) merges professional, interpersonal and instrumental competence, and distinguishes between ‘(inter)cultural’, ‘subject area’ and ‘psychological’ competences. Scarpa and Orlando (2017) also draw on the first holistic approach when applying the taxonomy of the EMT competence framework of 2009 (including translation service provision competence, language competence, intercultural competence, information mining competence, thematic competence and technological competence) to legal translation. In line with the QUALETRA project and Directive 2010/64/EU, they focus on the skills required for translating documents in criminal proceedings.

More recently, ISO 20771:2020 (*Legal translation – Requirements*) refers to the following (similar) competences: translation competence; linguistic and textual competence; specialist legal field competence; competence in research, information acquisition and processing; legal culture competence; and technical competence^3^ (ISO 2020, 8–9) (see a comparison of models in Table 1). As suggested by Monika Popiołek (2020), project leader for ISO 20771:2020, ‘the legal translator competences set in ISO 20771 include similar competence components’ as those described in Prieto Ramos (2011) (Popiołek 2020, 30). She considers that this approach ‘includes all the relevant components’ (ibid 26) and is ‘quite forward thinking’ (ibid 27), and that our ‘argument that the integral development of legal translation competence requires specific interdisciplinary methodologies and needs to be process-oriented, focus on the legal translation-specific know-how, a combination of practical translation skills and legal knowledge and adhere to a rigorous translation process, reflects some of the thinking behind the legal translator competence requirements set in the ISO 20771 standard as well’ (ibid 27).

**Table 1.** A comparison of LTC components in recent approaches

Prieto Ramos (2011)	Piecychna (2013)	Soriano Barabino (2016)	Scarpa and Orlando (2017) (EMT 2009 grid)	ISO 20771:2020
Strategic or methodological competence	Psychological competence (including strategic and service provision components)	Strategic competence	Translation service provision competence - interpersonal dimension - production dimension	Translation competence
Interpersonal and professional management competence		Professional, interpersonal and instrumental competence		---
		Psychological competence		
Communicative and textual competence	Linguistic competence	Communicative and textual competence	Language competence	Linguistic and textual competence in the source language and the target language
	Textual competence			
Thematic and cultural competence	Thematic competence	Subject area competence	Thematic competence	Specialist legal field competence
		(Inter)cultural competence	Intercultural competence -sociolinguistic dimension - textual dimension	Legal culture competence
Instrumental competence (documentation and technology)	---	*(integrated in professional competence above)*	Information mining competence	Competence in research, information acquisition and processing
			Technological competence	Technical competence

Popiołek points to ‘the focus on legal domain competence […], professional practice and the legal translation process orientation’ as the ‘most notable similarities between ISO 20771 and Prieto Ramos’s approach’ (ibid 33), and then highlights some differences that derive from the divergent scope and purpose of an ISO standard for translation service provision, as opposed to a descriptor of LTC and a process-oriented methodology for developing this competence. In her comparison, Popiołek confuses (1) the translation decision-making process summarised in Prieto Ramos (2011), where self-revision is a key stage in any translation job, with (2) revision by another translator or reviser as a workflow requirement for quality assurance that falls outside the scope of a competence descriptor for individual translators. Workflow specifications for service provision are not developed in LTC models, which are aimed at describing the skills required of individual translators. Of course, these may include revision competence as part of the core translation competence, for example, ‘quality control’ (i.e. revision but also review or other quality checks) as part of strategic or methodological competence in Prieto Ramos (2011). The same caveat applies to specific qualifications recommended in ISO standards, which deserve specific attention and are not discussed in competence descriptors (see discussion in Prieto Ramos and Guzmán 2024).

ISO 20771:2020 refers to legal translation in institutional settings in its Annex B, which highlights the relevance of legal translation in multilingual national and international organisations. However, the **competences required in institutional translation** more broadly remain under-researched. To date, the majority of descriptions of institutional translator profiles, mostly put forward by practitioners (e.g. Wagner, Bech, and Martínez 2012; relevant chapters in Borja Albi and Prieto Ramos 2013; Mavrič 2022), focus on specific institutions, but do not propose, apply or test a structured translator competence model across organisations.

The most relevant work on the subject thus far has been carried out by the UN’s Anne Lafeber (2012, 2022). The author conducted two surveys, in 2010 and 2021, to examine the extent to which institutional translation professionals had changed their views about the impact of specific skills on translation quality and the skills that are most often lacking among new recruits. The list of 39 skills and types of knowledge in the first survey, based on a review of the literature on institutional translation and translator competence, was expanded to 51 items in 2023 to reflect the latest trends in the field.

Knowledge of the source language, knowledge of punctuation and spelling rules in the target language and the ability to ‘make effective use of recycled content within a computer-assisted translation environment (text generated by translation memory software, concordance searches)’ (Lafeber 2022, 35) were among the high-impact and commonly found skills, while understanding obscure passages and complex topics, conveying nuances, ensuring coherence and producing idiomatic translations (Lafeber 2022, 34) were among the high-impact but most often lacking skills. The most significant increases in the impact ratings since 2010 were related to using recycled content efficiently and subject-matter knowledge (Lafeber 2022, 37); and the most marked increases in oft-lacking skills were related to thematic or domain competence: knowledge of the organisation, subject-matter knowledge and mastering new subjects quickly (Lafeber 2022, 39).

In another survey conducted in 2021, Froeliger et al. (2022) asked a more limited number of institutional translators^4^ how they perceived the relevance of the updated EMT competence framework (EMT 2017) for their work. The study provides clear evidence of the inadequacies of this framework to describe translator competence in institutional settings. A total of 13 out of 35 skills listed in the EMT descriptors were rated below 6 in a scale of 0 to 10, and only 10 reached a score of 8 or higher. These highest scoring skills included five out of 14 skills within ‘translation’ competence, three out of six within ‘personal and interpersonal’ competence, two out of six within ‘technology’ competence, and none of the nine skills included in the ‘service provision’ competence section (Froeliger, Krause, and Salmi 2022, 23). The top score overall went to ‘Comply with deadlines, instructions and specifications’ (9.40), which is included under ‘personal and interpersonal competence’, as opposed to other models in which time management and compliance with guidelines and specifications are generally associated with service provision or professional management. These skills, which are a must for any professional translator, were rated based on their general relevance for translation work, but not from the perspective of impact on translation quality as in Lafeber (2022). As a result of the abovementioned highest score, the ‘personal and interpersonal’ competence of the EMT framework registered the top average rating (7.35), even higher than translation competence (6.98).

In fact, in this framework, the unbalanced fragmentation of competence components leads to methodological flaws and a distorted picture of the most relevant competences required in institutional translation settings. For example, irrelevant skills such as ‘Use social media responsibly for professional purposes’ (score of 3.85) or ‘Approach existing clients and find new clients’ (3.30) are proportionally assigned more weight for the average rating of the relevant competence than the most relevant core translating and (self-)revising skills within translation competence. Additionally, language competence is excluded from the study, and thematic or subject area competence,^5^ a key component of specialised translation competence in all expert models (see above) and in the 2009 version of the EMT framework, is diluted as one of 14 skills within ‘translation competence’ in the 2017 EMT framework, contributing further to the inadequacy of the framework.

The framework does not refer to ‘specialised’ content or translation in any other component, and areas such as legal or financial translation, which comprise top-end segments of the translation market (and important areas in institutional translation), are not mentioned either. However, in describing the skill ‘Translate and mediate in specific intracultural and intercultural contexts’, the choice of examples provided (‘public service translation (and interpreting), website or video-game localisation and accessibility, community management, etc’.) most probably explains the borderline score obtained by this component among institutional translators (5.70).

Due to the arbitrary fragmentation of competences and the problematic merging of strategic and thematic competences under ‘translation (strategic, methodological and thematic competence)’ (EMT 2017, 7), the high relevance of thematic competence actually goes unnoticed in this approach. The relevance score registered by ‘thematic and domain-specific knowledge’ in the survey is high (8.36), but as a result of the abovementioned fragmentation, this component is valued less than ‘using social media responsibly’ (with a relative weight of 0.64 within the personal and interpersonal competence average of 7.35, *versus* a relative value of 0.60 of thematic and domain-specific knowledge within the translation competence average of 6.98).

The downgraded status of thematic competence in the 2017 version of the EMT framework remains a significant weakness. Its inconsistency with professional requirements, apparently for the sake of including more diverse MA programmes within the EMT (according to the network managers^6^), introduces a gap between EMT requirements for training programmes and professional expectations, as evidenced by a growing wealth of research, standards and surveys (see above-mentioned studies on translator competence, Esfendiari et al. 2019 and Popiołek 2020,^7^). The latest 2022 (minor) revision of the framework (EMT 2022) was a missed opportunity to redress this distortion. The survey results outlined in Froeliger et al. (2022) further highlight the need for a more tailored competence framework for institutional translation.

## 3. Towards an updated competence model for institutional translation

In this section, we will describe how our original LTC model has been tested and adapted to reflect the broader scope of institutional translation. The revision of the descriptors was based on the triangulation of a wealth of data gathered as part of the LETRINT project.

The first preliminary step consisted of verifying the relevance of LTC in institutional translation settings by examining the volumes and genres of translated texts in several EU and intergovernmental organisations (IGOs) (Prieto Ramos and Guzmán 2021), and by crossing this data with those obtained in a survey of institutional translators (from 12 organisations) (Prieto Ramos 2020) and in 33 structured interviews with 45 service heads and quality advisers. The correlative analysis of this data corroborated that legal (and administrative) translation in a broad sense (including law- and policy-making, implementation monitoring, adjudication and administrative functions) is the most prominent commonly-shared translation specialisation at international organisations from a quantitative and a qualitative perspective. It accounts for a significant proportion of multilingual text production and permeates institutional communication more generally, as all activities must be consistent with the legal framework and procedures of each institution.

Overall, the evidence obtained through this multi-faceted mapping confirmed the relevance of the legal translation paradigm for institutional translation. As in law and legal communication, institutional discourses must convey the intended message accurately and conform to the applicable legal framework, institution-specific conventions and the relevant precedents in institutional work. Also, like law, institutional missions and policies cover a myriad of subject matters, as reflected in the high degree of terminological hybridity of institutional text genres (Prieto Ramos and Cerutti 2022). This further supports the relevance of LTC in institutional translation settings, as LTC also entails the ability to research the range of subjects addressed by legal texts and proceedings, encompassing not only legal meaning, but also economic, technical or scientific topics, for example.

In a subsequent phase of the project, the same LTC model was used to analyse the competence requirements found in 224 vacancy notices for 290 competitions for translator and reviser positions of several organisations of varying sizes and domain specialisations between 2005 and 2020 (Prieto Ramos and Guzmán 2022). The vacancy components related to skills and knowledge types fit within the five sub-competence categories of the original model. The core language and strategic or methodological competences were consistently found in all the notices, while skills or knowledge associated with thematic, instrumental and interpersonal and professional management sub-competences (including several soft skills) featured explicitly in an average of 78%-88% and 86%-98% of notices for translator and reviser positions, respectively, per setting.

The high relevance of thematic competence is also acknowledged by the fact that most institutions accept a degree in any or several fields of study as pathways for recruitment, except for the Court of Justice of the EU (CJEU), where a law degree is required to be appointed as a lawyer-linguist and work as a translator. Otherwise, translation is the most commonly preferred field of study, followed by languages and law, which again attests to the relevance of legal knowledge for institutional translation. Technical or scientific fields or economics are also listed for certain positions (e.g. for WIPO’s patent translation positions and the WTO, respectively), in line with each institution’s main area of work.

Apart from confirming the relevance of the sub-competence categories proposed in 2011, the comparative and diachronic examination of job descriptors and requirements provided an invaluable source for adapting the competence model to current practices and expectations in institutional translation, including the gradual integration of technological skills. For example, terminology management and revision work were often mentioned in notices, although the latter was reserved for reviser positions in the case of the UN system; and the use of computer-assisted translation (CAT) tools was increasingly required. This trend was more systematic in the case of institutions such as the WTO and WIPO, which also took the lead in referring to post-editing in job descriptors for patent translation since 2015.

Finally, five additional interviews were conducted with translation managers from several institutions between 2021 and 2022 to update the information gathered in the first round of interviews, particularly with regard to the integration of neural machine translation (NMT) in work procedures. This was further enriched with insights provided by numerous translation professionals working in or for international organisations through more informal exchanges.

The resulting revised descriptor of legal and institutional translator competence includes the following interrelated components:

1. *Strategic or methodological competence*: controls the application of the other competences and comprises: analysis of translation briefs and purposes, identifying and solving translation problems and, more broadly, implementing strategies or methods to achieve communicatively adequate translations, self-revise and justify translation decisions, and revise or post-edit texts to ensure the expected translation quality.
2. *Language or communicative and textual competence*: linguistic, sociolinguistic and pragmatic knowledge necessary to understand source texts and produce correct and idiomatic target texts in diverse communicative situations, including knowledge of linguistic variants, registers, terminology, specialised language uses and genre conventions.
3. *Thematic and cultural competence*: ability to apply specialised and cultural knowledge (e.g. knowledge of legal systems or traditions, legal sources and concepts in the case of legal content) in order to understand and accurately convey specialised meaning or cultural references in translation or revision processes.
4. *Instrumental competence*: knowledge of specialised sources and terminology management tools, efficient use of relevant and reliable resources for information mining, and application of computer tools in translation and revision tasks, including information technology software and machine translation systems.
5. *Interpersonal and professional management competence*: time management, teamwork, efficient adaptation to the working environment (including ergonomic requirements), and ability to interact with clients, recipients or requesting officers and other professionals, in line with applicable working guidelines and ethical standards.

**Figure 1.**
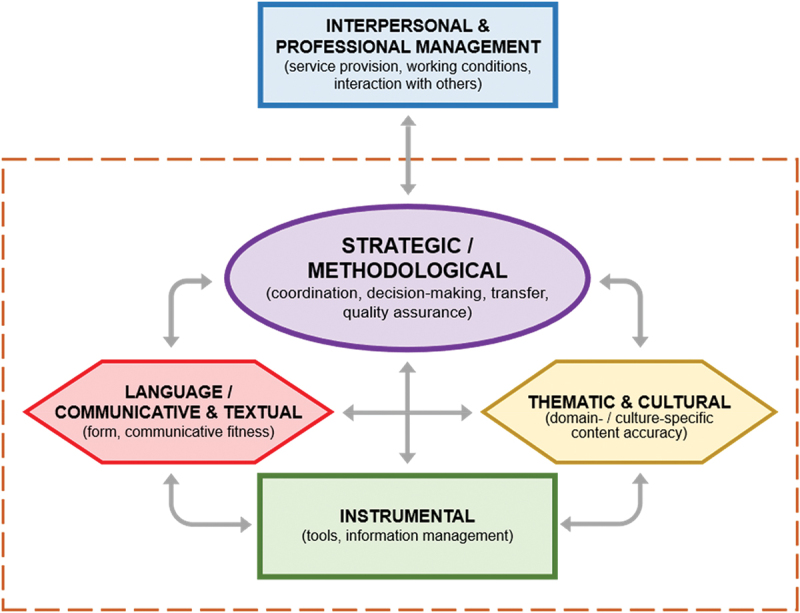
Representation of updated translator competence descriptor.

As highlighted in the first LTC descriptor, all the sub-competences must be understood dynamically rather than as isolated components. They are all interconnected under the coordination of strategic or methodological competence, which remains ‘the engine that makes the whole translation machinery work’ (Prieto Ramos 2011, 14). It focuses on analysing the elements and triggering the translational and supporting actions needed for decision-making to ensure communicative adequacy. This entails employing language competence (focus on textual form and communicative fitness) and thematic or cultural competence (focus on accurate domain- or culture-specific content) drawing on the efficient assistance of instrumental competence (focus on tools and information management) and applying interpersonal and professional management competence to adapt the entire process to the relevant working conditions and service provision requirements (see Figure 1). As an example of the interconnection between sub-competences, knowledge of specialised language and sources in a particular domain can be critical for the efficient use of tools, e.g. in defining searches and assessing data relevance and reliability for a translation. At the same time, genre conventions and terminology, for instance, are inextricably linked with linguistic and thematic knowledge.

While dissecting the competence components can be particularly beneficial for developing the relevant skills and knowledge to improve performance (especially in training contexts), experience and specialisation usually contribute to automating certain actions and decision-making strategies, and to the growing integration of sub-competences (see, e.g. Kaiser-Cooke 1994). As noted by Kiraly (2013, 208–209), in a highly experienced translator’s ‘translatory moment’, ‘the potential links between nodes in the network [of sub-competences] are innumerable and unpredictable and the decision-making processes uniquely adapted to each new translation problem’.

The updated description of sub-competence categories remains unchanged from the original LTC descriptor, except for the explicit reference to language in ‘language or communicative and textual competence’ for further clarity (see comparative Table including the original LTC descriptor and the updated descriptor in the Appendix). Based on the supporting research conducted, the fragmentation or merging of categories was deemed unnecessary. For example, splitting thematic and cultural competence would appear somewhat arbitrary, since domain-specific content is often culture-bound (e.g. knowledge of national administrative structures or technical bodies, or legal notions and procedures characteristic of specific legal cultures). In fact, the distinction between ‘specialist legal field competence’ and ‘legal culture competence’ in ISO 20771:2020 (ISO 2020, 8–9) is rather redundant, as it is based on overlapping definitions (in particular, the ‘ability to understand specialist legal content’ in the first definition and ‘understanding of legal procedures and systems’ in the second).

The same holds true for the distinction between information mining or processing and technical or technological competences in other models (see Table 1 above), as they are two sides of the same coin (instrumental competence) and cannot be dissociated from each other in the light of current professional practices. In contrast, psychological components such as self-motivation and self-confidence (e.g. Soriano Barabino 2011, 149, drawing on Kelly 2005), as well as several soft skills underpinning any professional service provision, are implied by other competences, particularly interpersonal and professional management competence.

As for the more specific descriptor revisions, *strategic or methodological competence* was maintained as the central overarching competence coordinating the application of all the others, as they interact for translator decision-making and service provision. The key elements, however, were updated to accommodate all approaches and taxonomical preferences in connection with translator strategies or methods to achieve communicative adequacy, while also making more explicit reference to quality control actions and quality assurance in general, including revision and post-editing, in line with our preliminary findings. More specifically, instead of ‘implementation of transfer strategies (translation procedures)’, ‘decision-making argumentation’ and ‘quality control’ (Prieto Ramos 2011, 12), the updated descriptor refers to ‘implementing strategies or methods to achieve communicatively adequate translations, self-revise and justify translation decisions, and revise or post-edit texts to ensure the expected translation quality’.

This also reflects a trend towards increasingly nuanced approaches to fit-for-purpose quality in translation service provision, as well as the hybridisation of translation processes with more diverse inputs than a decade ago, including edits or rewriting of translation memory matches or MT suggestions (see also next section). While these actions require specific operational knowledge and skills and can be the subject of dedicated descriptors,^8^ they are closely intertwined and share the core strategic or methodological proficiency to achieve communicative adequacy of the final product. In this kind of comprehensive approach, the use of ‘strategic or methodological competence’ rather than ‘translation competence’ is a deliberate choice, not only to avoid the risk of reductionism or confusion between ‘translation macro-competence’ and the core ‘translation sub-competence’ (strategic or methodological, as understood here), but also to highlight the multiple facets and versatility of translator competence, beyond translation tasks *stricto sensu*.

As regards the other competences, the updated version of *language or communicative and textual competence* includes a more explicit reference to the use of linguistic knowledge ‘to understand source texts and produce correct and idiomatic target texts’ as paramount quality requirements. In the description of *instrumental competence*, the revision involved eliciting the ‘efficient use of relevant and reliable resources for information mining’, an aspect emphasised in the preliminary research, as well as specifying ‘information technology software and machine translation systems’ within the ability to use ‘computer tools in translation and revision tasks’.

Likewise, *interpersonal and professional management competence* was expanded to include components that apply to all professional translators in any specialisation, and were previously taken for granted: ‘time management’ and ‘efficient adaptation to the working environment (including ergonomic requirements)’, both important aspects of working conditions in institutional settings. In addition, the interaction with clients and other professionals was revised to specify ‘clients, recipients or requesting officers and other professionals’ in accordance with the wide range of actors mentioned by institutional informants. Adherence to ‘working guidelines’ was also accordingly elicited, while the original reference to ‘fiscal obligations’ was considered less relevant and was finally excluded.

Last but not least, in line with the broader scope of institutional translation, the most distinctive legal domain elements were reformulated to include other specialised areas (e.g. ‘specialised language uses and genre conventions’ rather than ‘specialised legal linguistic uses and legal genre conventions’ within language or communicative and textual competence), or were kept as relevant examples in the case of *thematic and cultural competence*, but not as the exclusive focus: ‘ability to apply specialised and cultural knowledge (e.g. knowledge of legal systems or traditions, legal sources and concepts in the case of legal content) in order to understand and accurately convey specialised meaning or cultural references in translation or revision processes’.

Finally, it is relevant to emphasise at this point that, as in the case of the original LTC model, the revised descriptor is not intended to outline a prototypical translation workflow or to offer an exhaustive list of all the skills and knowledge required. Rather, it is conceived to group together, in a balanced way, the most relevant competence components expected of any individual translator to work professionally in legal translation and other areas of specialised translation, especially in institutional settings but not exclusively, according to the core commonalities identified in our research.

## 4. Examining the relevance of translator competence components in institutional translation settings

The revised descriptor was put to the test through a survey in which translation professionals from international organisations were asked about the relevance of the various sub-competences. The survey was distributed through translation service managers of IAMLADP (the International Annual Meeting on Language Arrangements, Documentation and Publications, the world’s largest network of institutional language services) in the autumn of 2022, and remained open until mid-February 2023.

A total of 474 translation professionals from 24 organisations, including a sufficiently representative number of EU institutions and intergovernmental organisations (IGOs), took part in the survey. The highest proportion of participants were from the largest translation services (the European Commission, the European Parliament, the Council of the EU, the CJEU, and the United Nations (UN), with between 40 and 77 respondents each), followed by medium-sized services (the European Court of Auditors, the joint services of the Committee of the Regions (CoR) and the European Economic and Social Committee (EESC), the World Intellectual Property Organization (WIPO), the World Trade Organization (WTO), the European Central Bank (ECB) and the European Investment Bank (EIB), with between 12 and 23 respondents each). A total of 31 freelancers (6.54% of respondents) also qualified for the survey, as they worked primarily for international organisations onsite or remotely, with temporary contracts or regular assignments.

In order to compare the perceptions of relevance of competence components for legal and institutional translation depending on the participants’ profiles, respondents were asked about their academic background and main translation specialisations, and about their experience in legal and institutional translation. Their profiles were classified into several clusters:

- LL (translation practitioners holding a degree in law, including those who are not formally appointed as ‘lawyer-linguists’)
- LT (those with a degree in translation and some training in law or legal translation)
- T (translators or revisers holding a degree in translation but with no training in law or legal translation)
- T0 (translators or revisers with other backgrounds not included in any of the above groups)

Responses indicated that the main area of specialisation of translation practice was legal or administrative (overall average of 77.43%), followed by economic or financial (39.66%) and technical or scientific (32.49%) (see Table 2). These results align with those of our previous survey (Prieto Ramos 2020) in pointing to the frequent combination of legal or administrative translation with one or more additional thematic fields, which varied across institutions. As in the previous study, economic or financial translation was more frequent than technical or scientific translation, for example, at the WTO, the Council of the EU or the European Parliament, and it was even more prominent than legal or administrative translation at financial institutions (e.g. the ECB, the EIB or the OECD). However, technical or scientific translation was the second most common specialisation at the UN or the European Commission, and the most prevalent at WIPO.

**Table 2.** Main areas of translation specialisation (distribution across institutions of affiliation of at least five respondents).

	Legal/Administrative	Economic/Financial	Technical/Scientific	Other
**EU institutions**	**79.17%**	**40.48%**	**25.60%**	**24.70%**
Council of the EU*	84.42%	48.05%	31.17%	36.36%
Court of Justice of the EU	98.55%	10.14%	2.90%	8.70%
European Central Bank	91.67%	100%	-	8.33%
European Commission	73.58%	33.96%	39.62%	22.64%
European Court of Auditors	56.52%	82.61%	26.09%	21.74%
European Investment Bank	58.33%	66.67%	33.33%	16.67%
European Parliament	74.55%	34.55%	23.64%	25.45%
Joint Services of CoR and EESC	46.67%	46.67%	40%	40%
Translation Centre for the Bodies of the EU (CdT)	83.33%	41.67%	50%	25%
**IGOs**	**69.16%**	**37.38%**	**52.34%**	**21.50%**
European Patent Office	100%	20%	20%	40%
ILO	100%	20%	20%	20%
OECD	33.33%	83.33%	16.67%	16.67%
United Nations	67.50%	32.50%	57.50%	27.50%
WIPO	33.33%	-	66.66%	-
WTO	94.44%	72.22%	33.33%	11.11%
Other	61.90%	33.33%	66.66%	23.81%
**Freelancer (several institutions)**	**87.10%**	**38.71%**	**38.71%**	**25.81%**
Total**	**77.43%**	**39.66%**	**32.49%**	**24.05%**

*Relative value = % of participants from the same institution.

**Relative value = % of all participants (*n*=474).

As for academic and professional backgrounds (see Table 3), all profiles were well represented. Unsurprisingly, translation graduates made up the largest group (54.01%), including 35.23% of LTs and 18.78% of Ts, followed by LLs (24.68%, including 100% at the CJEU) and T0 translators (21.31%). Apart from the CJEU specificity, the most remarkable difference between EU institutions and IGOs was found in the proportions of LT and T0 profiles (52.34% and 10.28%, respectively, at IGOs *versus* 34.83% and 32.21% at EU institutions excluding the CJEU).

**Table 3.** Distribution of profile groups per institution type and among freelancers.

	LL	LT	T	T0
EU institutions (excl. CJEU)*	11.61%	34.83%	21.35%	32.21%
Court of Justice of the EU	100%	-	-	-
IGOs	10.28%	52.34%	27.10%	10.28%
Freelancers (several institutions)	19.35%	58.06%	9.68%	12.90%
Total**	24.68%	35.23%	18.78%	21.31%

*Relative value = % of participants from the same institution.

**Relative value = % of all participants (*n*=474).

**Figure 2.**
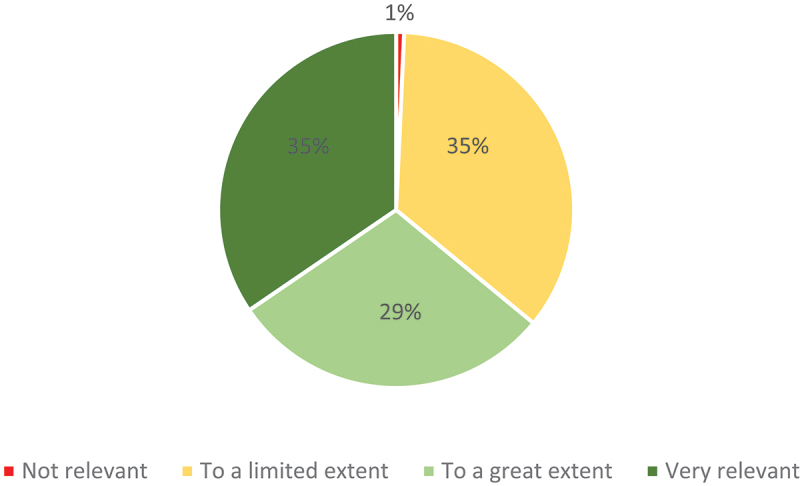
Relevance of legal or institutional translation training (81 training institutions).

**Figure 3.**
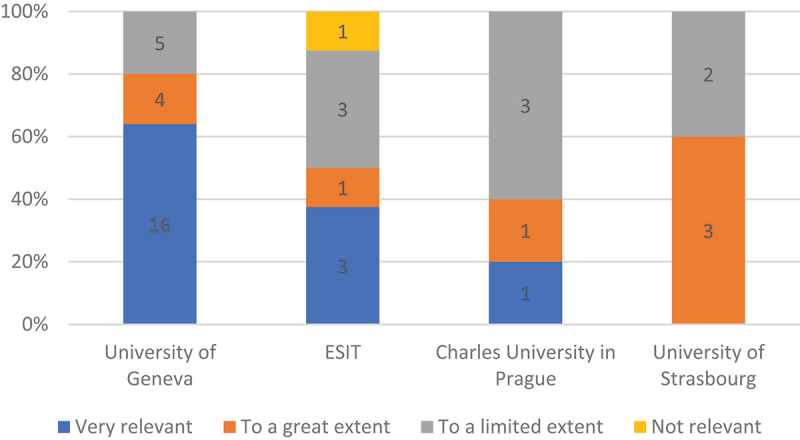
Relevance of legal or institutional translation training (training institutions of at least five respondents).

When asked specifically whether and where they had received training in legal or institutional translation prior to their professional practice, 139 respondents (29.32%) replied affirmatively, and 81 training institutions were mentioned. However, only four universities received five or more replies, significantly led by the University of Geneva (25 or 18% of all positive replies), and followed by ESIT (8), Charles University in Prague (5) and the University of Strasbourg (5). The perceptions of relevance of such training for professional practice in institutional settings varied enormously. While the average for all programmes revealed a relative distribution of one third each for ‘very relevant’, ‘to a great extent’ and ‘to a limited extent’, the University of Geneva scored significantly higher than the rest (see Figures 2 and 3) with almost two thirds of ‘very relevant’ views.

**Figure 4.**
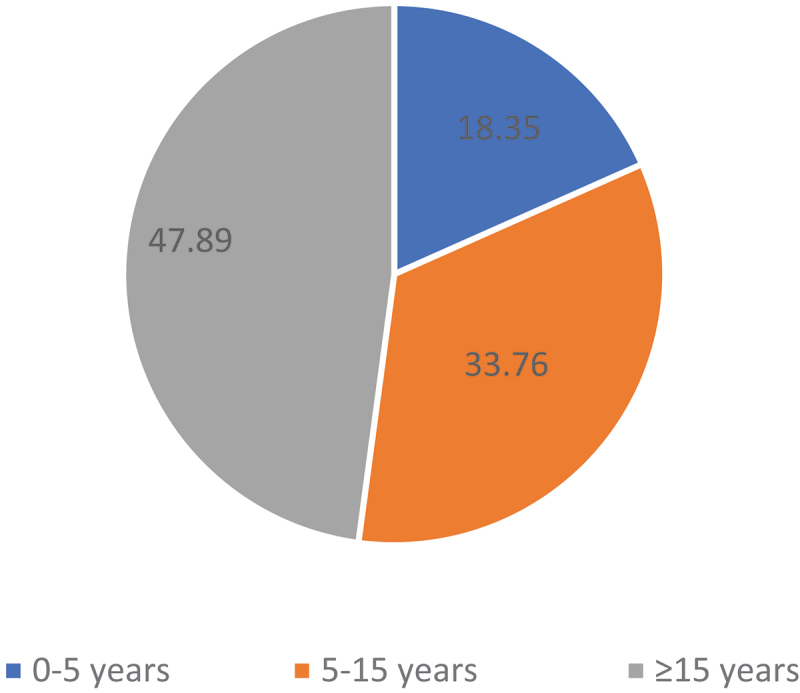
Years of experience in institutional translation.

In terms of experience, the T0 group seemed to be the most senior. It registered the longest experience in translation and revision in general, but not in legal translation (see Table 4). This can probably be explained by recruitment practices before the proliferation of university programmes in translation. The pattern of less frequent legal translation practice is even more marked in the second most experienced group overall, that of Ts, while LTs and especially LLs stood out for their track record in legal translation. The average years of experience for all the groups range between 11.46 of translation and 9.08 of revision at their current institutions, and 18.68 and 13.49 years of total translation and revision experience, respectively. The distribution of respondents according to their experience in institutional translation offers a very interesting snapshot of the composition of translation staff at international organisations, with very similar results across the board: almost half of respondents had 15 or more years of experience, one third stated between 5 and 15, and 18.35% of respondents had up to five years of experience (see Figure 4).

**Table 4.** Average years of translation and revision experience per profile group.

		LL	LT	T	T0	Average
At current institution	Translation	11.98	10.34	11.96	12.26	11.46
	Revision	9.01	7.58	9.59	11.22	9.08
Institutional translation	Translation	13.24	13.44	13.79	15.39	13.87
	Revision	9.42	9.30	9.94	13.05	10.25
Legal translation	Translation	14.41	12.01	7.51	11.34	11.61
	Revision	10.10	9.04	5.66	9.55	8.77
Overall	Translation	17.16	18.29	19.84	20.06	18.68
	Revision	12.30	12.97	14.09	15.22	13.49

This diverse population of institutional translation professionals were asked to assess whether the revised breakdown of competences presented above summarises what is required to translate in their professional setting, and to evaluate the relevance of each competence to ensure translation quality based on their experience. They were able to reply ‘yes’, ‘no’ or ‘only partially’ to the first question, and were invited to comment on what they would ‘add or change’ in case of a negative or a partially positive answer.

The descriptor was validated by an overwhelming majority of almost 98% of respondents, with no significant variations according to profile or years of experience (Table 5). Among the valid ‘only partially’ (9) and ‘no’ (1) replies, the main reasons given were the preference to expand or rephrase specific categories, particularly to make some skills more explicit (e.g. ‘critical thinking to be able to detect possible mistakes’, ‘logical reasoning skills’ or ‘consistency competence’) or to further emphasise their relevance, even if they were included in the descriptor (especially legal and other subject matter knowledge, and productivity or deadline requirements). After an in-depth analysis of these comments, no modification of the overall breakdown of competences was deemed necessary.

**Table 5.** Approval of adapted competence descriptor per profile group and years of experience.

	Yes	Only partially	No
Profiles			
LL*	98.29%	1.71%	-
LT	97.60%	2.40%	-
T	96.63%	2.25%	1.12%
T0	99.01%	0.99%	-
Experience			
0–5 years*	98.85%	1.15%	-
5–15 years	95.63%	4.38%	-
≥15 years	99.11%	0.44%	0.44%
Overall**	97.89%	1.90%	0.21%

*Relative value = % of participants within group.

**Relative value = % of all participants (*n*=474).

A specific question was asked to test the deliberate inclusion of post-editing, together with revision, within strategic or methodological competence, and more particularly, whether translation professionals considered these actions as similar processes calling for essentially the same core decision-making competence to ensure translation quality. 94.50% validated affirmatively, including 72.21% of replies pointing to partial similarity and 22.29% of views as very similar competences.

**Figure 5.**
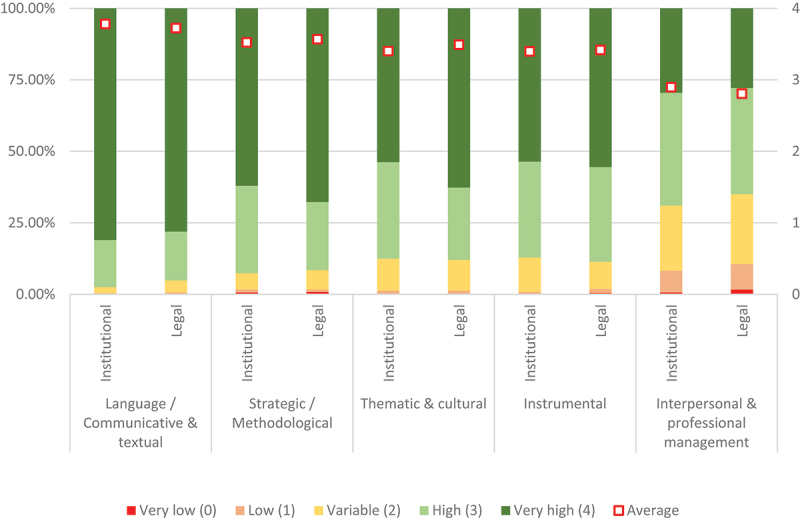
Competence relevance to ensure translation quality (overall scores).

As regards the relevance of each competence to ensure translation quality, a double question was asked to compare the relevance for institutional translation in general and for the translation of ‘texts of a legal or administrative nature, either binding or non-binding (e.g. legal acts, treaties, agreements, resolutions, model laws), or texts about compliance with rules, guidelines or obligations (e.g. documents used in monitoring procedures or court proceedings)’. Overall, on a scale between 0 (very low relevance) and 4 (very high relevance), all competences were rated between 3.40 and 3.78 (i.e. as highly or extremely relevant), except for interpersonal and professional management competence, which scored an average of just under 3 (see Table 6 and Figure 5). These results corroborate the suitability of the translator competence descriptor for the survey population.

**Table 6.** Competence relevance to ensure translation quality.

Competence	Translation	Very low (0)	Low (1)	Variable (2)	High (3)	Very high (4)	Average
Language/Comm. & textual	Institutional	-	2 (0.42%)	10 (2.11%)	78 (16.46%)	384 (81.01%)	3.78
	Legal	1 (0.21%)	2 (0.42%)	20 (4.22%)	81 (17.09%)	370 (78.06%)	3.72
Strategic/Methodological	Institutional	3 (0.63%)	5 (1.05%)	27 (5.70%)	145 (30.59%)	294 (62.03%)	3.52
	Legal	4 (0.84%)	4 (0.84%)	32 (6.75%)	113 (23.84%)	321 (67.72%)	3.57
Thematic & cultural	Institutional	-	6 (1.27%)	53 (11.18%)	160 (33.76%)	255 (53.80%)	3.40
	Legal	1 (0.21%)	5 (1.05%)	51 (10.76%)	120 (25.32%)	297 (62.66%)	3.49
Instrumental	Institutional	1 (0.21%)	3 (0.63%)	57 (12.03%)	159 (33.54%)	254 (53.59%)	3.40
	Legal	2 (0.42%)	7 (1.48%)	45 (9.49%)	157 (33.12%)	263 (55.49%)	3.42
Interpersonal & professional management	Institutional	3 (0.63%)	36 (7.59%)	108 (22.78%)	187 (39.45%)	140 (29.54%)	2.90
	Legal	8 (1.69%)	42 (8.86%)	116 (24.47%)	176 (37.13%)	132 (27.85%)	2.81

The highest scores were obtained by language or communicative and textual competence, strategic or methodological competence, and thematic and cultural competence, closely followed by instrumental competence. The most marked differences between the averages for institutional translation in general and legal translation in particular were those registered for thematic and cultural competence (+0.09 for legal translation) and interpersonal and professional management competence (+0.09 for institutional translation in general). Legal translation also scored higher in the case of strategic or methodological competence (+0.05) and instrumental competence (+0.02). These differences can be associated to the specificities of legal analysis, transfer procedures and knowledge of legal sources required for legal translation. However, language competence scored higher for institutional translation in general (+0.06).

**Figure 6.**
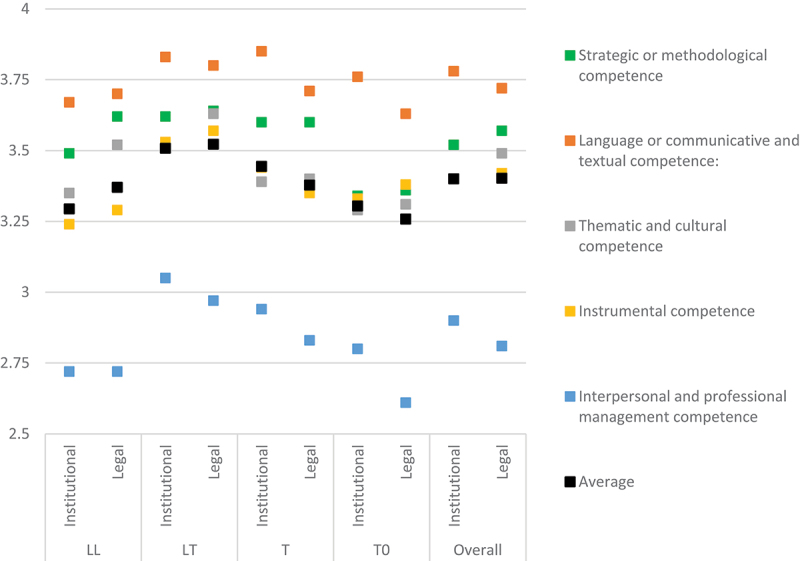
Competence relevance to ensure translation quality (scores per profile group).

These patterns generally apply to all profile groups and experience levels, with strategic and thematic competences perceived as more relevant for translating legal texts in particular than for institutional translation more broadly among all groups (except for equal values for strategic competence among T translators), and higher values for interpersonal and professional management competence for institutional translation practice in general (or equal values in the case of LLs) (see Table 7 and Figure 6).

**Table 7.** Competence relevance to ensure translation quality (scores per profile group).

		Language/communicative & textual	Strategic/methodological	Thematic & cultural	Instrumental	Interpersonal & professional management	Average
LL	Institutional	3.67	3.49	3.35	3.24	2.72	**3.29**
	Legal	3.70	3.62	3.52	3.29	2.72	**3.37**
LT	Institutional	3.83	3.62	3.51	3.53	3.05	**3.51**
	Legal	3.80	3.64	3.63	3.57	2.97	**3.52**
T	Institutional	3.85	3.60	3.39	3.44	2.94	**3.44**
	Legal	3.71	3.60	3.40	3.35	2.83	**3.38**
T0	Institutional	3.76	3.34	3.29	3.33	2.80	**3.30**
	Legal	3.63	3.36	3.31	3.38	2.61	**3.26**
Overall	**Institutional**	**3.78**	**3.52**	**3.40**	**3.40**	**2.90**	**3.40**
	**Legal**	**3.72**	**3.57**	**3.49**	**3.42**	**2.81**	**3.40**

The most significant deviations were found among those who were not trained in translation or law (T0) and those with a more limited experience in institutional translation. In the case of T0 respondents, the average score for instrumental competence was 0.07 higher than thematic and cultural competence, most probably due to the need for these translators to fill specialised knowledge gaps through more research and information mining. In contrast, the more specialised profiles from a legal translation perspective registered the most marked positive differences for legal translation when assessing the relevance of thematic and cultural competence (+0.17 among LLs and + 0.12 among LTs), and strategic or methodological competence (+0.13 among LLs). Remarkably, those trained in translation, and especially in legal translation, showed the clearest awareness of the relevance of all competences. LTs showed the highest and most balanced perceptions for both institutional translation and legal translation (averages of 3.51 and 3.52, respectively).

**Figure 7.**
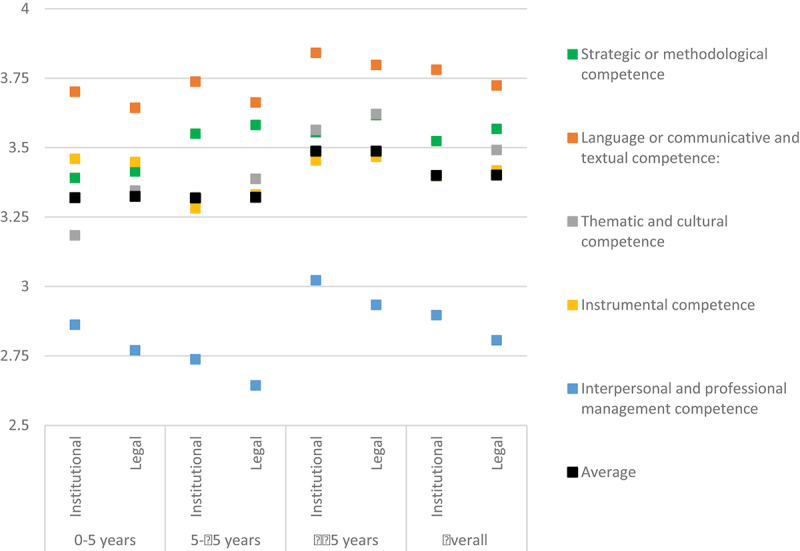
Competence relevance to ensure translation quality (scores per years of experience).

Those with the longest experience in institutional translation (15 years or more), regardless of their academic background, also showed the highest awareness of the relevance of the five competences under examination for institutional and legal translation (3.49 average, as opposed to 3.32 among the other groups) (see Table 8 and Figure 7). The most experienced group registered the highest values of the series for thematic and cultural competence (3.56 for institutional translation and 3.62 for legal translation), at the same level as strategic or methodological competence, and only below language competence (3.84 and 3.80, respectively, also the highest of the series for this competence). Interestingly, the differences between perceptions of competences for legal translation and institutional translation were more limited, generally under 0.08, when survey participants were grouped per years of experience.

**Table 8.** Competence relevance to ensure translation quality (scores per years of experience).

		Language/communicative & textual	Strategic/methodological	Thematic & cultural	Instrumental	Interpersonal & professional management	Average
0–5 years	Institutional	3.70	3.39	3.18	3.46	2.86	**3.32**
	Legal	3.64	3.41	3.34	3.45	2.77	**3.32**
5–15 years	Institutional	3.74	3.55	3.29	3.28	2.74	**3.32**
	Legal	3.66	3.58	3.39	3.33	2.64	**3.32**
≥15 years	Institutional	3.84	3.56	3.56	3.45	3.02	**3.49**
	Legal	3.80	3.62	3.62	3.47	2.93	**3.49**
Overall	**Institutional**	**3.78**	**3.52**	**3.40**	**3.40**	**2.90**	**3.40**
	**Legal**	**3.72**	**3.57**	**3.49**	**3.42**	**2.81**	**3.40**

The more junior group (up to five years of experience) registered the most remarkable difference between values for institutional and legal translation. They assigned +0.26 to the relevance of thematic competence for legal translation (3.34 *versus* 3.18 for institutional translation), thus acknowledging the need for specialised subject matter knowledge. Yet, the most notable deviation from the overall ranking of competence relevance was due to the high score obtained by instrumental competence, which was perceived by this group as the second most relevant to achieve translation quality, even greater than strategic or methodological competence. The relevance scores for instrumental competence among this group (3.46 for institutional translation and 3.45 for legal translation) are actually almost identical to those assigned by the most experienced group. However, our results point to a clear correlation between the accumulation of experience and increasing perceptions of relevance of the core language (+0.14 between early and late career groups), strategic (+0.17 for institutional translation and +0.21 for legal translation) and especially thematic (+0.38 and +0.28, respectively) competences. While all the values compared in this section remain within the high to very high relevance range, they are significant for identifying the potential impact of specialised training and experience on perceptions of translator competence.

## 5. Discussion and concluding remarks

The research presented here illustrates the empirical value of combining several sources of information from professional settings and triangulating this data as the basis to update a translator competence descriptor for subsequent validation. In this case, the evidence obtained through our preliminary mapping underscored the relevance of the legal translation paradigm for the analysis of institutional translation practices, both from a quantitative and a qualitative perspective. In turn, as reflected in our survey results, this field encompasses a great diversity of subjects and a wide range of academic and professional backgrounds among institutional translation professionals.

Our large-scale survey of such professionals has supported the timely adaptation of our 2011 LTC descriptor to cover all areas of institutional translation and accounting for the latest developments in working practices, including the integration of post-editing into the increasingly diverse translational actions within the scope of strategic or methodological sub-competence. This kind of updating exercise is essential to keep step with professional trends and enhance employability among translation graduates, especially considering the consolidated use of translator competence descriptors in curricular and syllabus design.

The survey results corroborate the relevance of five key sub-competences that encapsulate the skill-set and versatility expected of translators to adapt to multiple tasks, conventions, subjects, tools and professional requirements. Despite the recent hype about MT and the increasing importance of instrumental competence for the growing human-machine interaction in translation service provision, the perceptions of institutional translators and revisers generally point to the higher relevance of three core competences to ensure translation quality: language or communicative and textual competence, strategic or methodological competence (including post-editing), and thematic and cultural competence. Interpersonal and professional management competence ranked last, even if it was also highly valued.

Overall, the competence relevance ratings for institutional translation in general and legal translation in particular were within similar ranges, but strategic and especially thematic competences were perceived as more relevant in the case of the latter. This trend was particularly marked among the more specialised profiles from a legal perspective (LLs and LTs) and among respondents with a longer experience in institutional translation.

Those who had received more tailored training, including a translation degree and law or legal translation training (LTs), registered the highest awareness of competence relevance to ensure translation quality, while the reverse applied to translators who were not formally trained in translation, law or legal translation (T0s). A similar correlation was established between translation experience and increasing perceptions of relevance of core language, strategic and especially thematic competence. The relevance of instrumental competence was comparatively more highly valued than strategic and thematic competences only among the less experienced translators, but ranked similarly to the relevance score for the same competence among the most experienced group, thus debunking the belief that senior translation professionals are more adverse to technology.

In sum, these findings depict a clear connection between training in specialised translation and translation experience, and greater awareness of translation sub-competences to ensure product quality in general, as well as higher awareness of the most distinctive competence components in a specialised field such as legal translation. In turn, greater awareness of what is required to achieve translation quality is expected to be reflected in expert performance levels, something that deserves more empirical research (see Prieto Ramos and Guzmán 2024).

Our results are roughly in line with those obtained in two studies referenced above. In their survey of the private translation sector, Esfandiari et al. (2019) also found that ‘language competence’ and ‘thematic competence’ ranked at the top of the most relevant competences for professional practice. It thus seems that, regardless of market pressures driven by MT performance gains, translators across settings share a sense of what is required to achieve translation quality. And these competences comprise the most common weaknesses identified among the new recruits by the more experienced institutional translation professionals (Lafeber 2022). These gaps match the lower relevance rates for core language, strategic and thematic competences assigned by the less experienced respondents in our survey. Thematic competence, the one that registered the most marked deficit increases between 2010 and 2021 in Lafeber’s survey (2022), is also the one for which relevance perceptions to ensure translation quality seem to become more pronounced with translation experience according to our results.

Considering that today’s new generation of institutional translators is most often trained in translation, a combined reading of these findings leads us to a number of questions: are training programmes in translation sufficiently adapted to achieve excellence in specialised translation? By stressing (or perhaps over-stressing) the need to master technological tools, are the other core competences being over-shadowed or underestimated? Can this trend actually contribute to diminishing the relevance of human translators and translator training itself? Is it not becoming more compelling to focus on the persistent or emerging knowledge gaps and get back to the essence of what is required to gain the extra mile in terms of quality, also when interacting with artificial intelligence? Should this be accompanied by further education of stakeholders and the general public about why translation quality matters and what it takes to achieve it? In a landscape of increasing automation, substantive subject knowledge (or the ability to acquire it efficiently), together with excellent language and methodological skills, may well be gaining significance if the risks and tricks of machine input processing are to be successfully managed.

Indeed, the insights gathered through this research generate new momentum behind our call for further interdisciplinarity in specialised translator training (Prieto Ramos 2011, 18). The added value of subject matter knowledge and multi- or interdisciplinarity were also highlighted during the preliminary interviews of the LETRINT project. As recently expressed by Merit-Ene Ilja, from the European Commission’s Directorate-General for Translation, ‘university translation courses should ideally be interdisciplinary in nature and also cater for some domain knowledge’ (Ilja 2022, 217). As also revealed by the survey, this kind of interdisciplinary approach seems to bear fruit in light of training relevance perceptions among the respondents who graduated from the University of Geneva, the institution with the longest record in tailoring training to institutional translation needs (see also Way and Jopek-Bosiacka 2022).

The translator competence descriptor resulting from this research is the first of its kind specifically adapted to institutional translation needs on the basis of a comprehensive mapping of practices, and confidently validated by institutional translation professionals. Given the diverse text types and specialisations covered in these settings, the results can be extrapolated to other contexts of specialised translation. The descriptor and other findings can inform not only training ventures, but also professional appraisal, recruitment processes and translation service management. The results of this work can also be useful for other studies on translator competence, as illustrated by drawing on our data and approach as input for the development of a common template for level C (specialist translator) competence descriptors in Europe.^9^ Training institutions and stakeholders in the field can only benefit from further convergence and fine-tuning of approaches and models with evolving professional practices.

## Notes

1. For the sake of simplicity, ‘sub-competence’ and ‘competence’ will be used interchangeably to refer to the components of translator ‘competence’ as a ‘macro-competence’. Depending on the context or the approach, ‘translation competence’ may thus refer to this macrocompetence or to a core component or ‘sub-competence’ of overall translator competence.

2. ‘Legal Translation in International Institutional Settings: Scope, Strategies and Quality Markers’, led by the author (https://transius.unige.ch/letrint/).

3. This breakdown of competences differs from that of ISO 17100:2015 (*Translation services – Requirements for translation services*) in that the latter is not domain-specific and refers to ‘cultural competence’ and ‘domain competence’ more broadly (ISO 2015, 6).

4. Including 208 staff from international organisations and 129 from five national administrations in Europe, as opposed to over 1000 staff in the case of Lafeber (2022).

5. ‘Acquire, develop and use thematic and domain-specific knowledge relevant to translation needs (mastering systems of concepts, methods of reasoning, presentation standards, terminology and phraseology, specialised sources etc.)’ (EMT 2017 and 2022, 7).

6. Personal correspondence and exchanges as part of discussions to update the framework within an EMT working group on translation competence in 2017 and 2022.

7. According to Popiołek (2020, 21), ‘the EMT 2009 model was more structured and had a clearer distinction between competences which are frequently found in learning outcomes’. She criticises the fact that the 2017 framework amalgamates strategic, methodological and thematic sub-competences ‘to create a “super” translation competence, which does not allow for more detailed analysis and granularity’ (Popiołek 2020, 22). Esfandiari et al. (2019) used the 2009 model in their survey of 456 freelance translators with a minimum of five years of professional experience. They found that ‘language competence’, ‘thematic competence’ and ‘intercultural competence’ were considered the most needed for professional practice, followed by ‘translation service provision competence’, ‘information mining competence’ and ‘technological competence’.

8. On revision and post-editing competences, see e.g. Koponen, Mossop, and Robert 2021; Rico and Torrejón 2012; Robert, Remael, and Ureel 2017.

9. For more information, see the results of the European Strategic Partnership EFFORT (‘European Framework for Translation’): https://www.effortproject.eu/.

## Appendix

**Table A1.** Comparison of original LTC descriptor and the updated descriptor.

LTC descriptor (Prieto Ramos 2011)	Updated descriptor for legal and institutional translation (validated in 2022–2023)
Strategic or methodological competence: controls the application of the other skills and comprises: analysis of translation briefs, macro-contextualisation and general work planning, identification of problems and implementation of transfer strategies (translation procedures), decision-making argumentation, self-assessment and quality control.	Strategic or methodological competence: controls the application of the other competences and comprises: analysis of translation briefs and purposes, identifying and solving translation problems and, more broadly, implementing strategies or methods to achieve communicatively adequate translations, self-revise and justify translation decisions, and revise or post-edit texts to ensure the expected translation quality.
Communicative and textual competence: linguistic, sociolinguistic and pragmatic knowledge, including knowledge of linguistic variants, registers, specialised legal linguistic uses and legal genre conventions.	Language or communicative and textual competence: linguistic, sociolinguistic and pragmatic knowledge necessary to understand source texts and produce correct and idiomatic target texts in diverse communicative situations, including knowledge of linguistic variants, registers, terminology, specialised language uses and genre conventions.
Thematic and cultural competence: knowledge of legal systems, hierarchy of legal sources, branches of law and main legal concepts; awareness of asymmetry between legal notions and structures in different legal traditions.	Thematic and cultural competence: ability to apply specialised and cultural knowledge (e.g. knowledge of legal systems or traditions, legal sources and concepts in the case of legal content) in order to understand and accurately convey specialised meaning or cultural references in translation or revision processes.
Instrumental competence (documentation and technology): knowledge of specialised sources, information and terminology management, use of parallel documents, application of computer tools to translation.	Instrumental competence: knowledge of specialised sources and terminology management tools, efficient use of relevant and reliable resources for information mining, and application of computer tools in translation and revision tasks, including information technology software and machine translation systems.
Interpersonal and professional management competence: teamwork, interaction with clients and other professionals, knowledge of legal framework for professional practice and fiscal obligations, deontological aspects.	Interpersonal and professional management competence: time management, teamwork, efficient adaptation to the working environment (including ergonomic requirements), and ability to interact with clients, recipients or requesting officers and other professionals, in line with applicable working guidelines and ethical standards.
